# A Contribution to the Study of Joint Bodies from within Present in Articulations Otherwise Apparently Normal*Continued from March Number.

**Published:** 1915-05

**Authors:** Aime Paul Heineck

**Affiliations:** Chicago, Illinois


### TEXAS MEDICAL JURNAL

**Dr. F. E. Daniel, Founder. Established July, 1885**

MRS. F. E. DANIEL, - Publisher and Managing Editor

Published Monthly.—Subscription, $1.00 a Year.

VOL. XXX AUSTIN, MAY, 1915 NO. 11

The publisher is not responsible for views of contributors

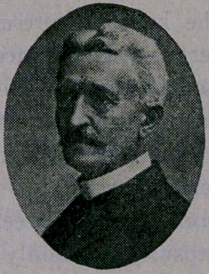

### Original Articles.

### A Contribution to the Study of Joint Bodies From Within Present in Articulations Otherwise Apparently Normal.*

BY AIME PAUL HEINECK, CHICAGO, ILLINOIS.

*Treatment.*—In all cases of joint-body herein considered, the involved articulation was opened. In almost all the cases one joint-body was removed; in the remaining cases two or more were extracted.

For the operative removal of joint-bodies we subject, in the absence of contraindications, our patients to general surgical anesthesia, employing either nitrous oxide-oxygen or sulphuric ether anesthesia, initiating the latter by nitrous oxide gas. General surgical anesthesia secures complete abolition of consciousness, complete muscular relaxation and more ample protection against pain.

For the removal of joint-mice, select an incision giving easy access to the joint-body or bodies present and inflicting a minimal amount of trauma upon the peri-articular structures. The position of the incision is largely determined by the location of the body or bodies to be removed. It is generally longitudinal. Joint may be opened directly over mouse. For the removal of knee joint-bodies some operators make an incision to the inner side of the patella, some to the outer side.

We advise that articulations be opened by longitudinal incisions, incisions parallel to the long axis of the limb, and in suitable cases, made directly over the joint-body itself. In the knee, make

*Continued from March Number.

your incision, if not directly over the joint-body itself, to the inner or to the outer side of the patella. On the inner side of the knee are present the thick muscular fibres of the vastus internus. If adapted to the case at hand, an incision external to and above the patella, parallel with the fibres of insertion of the vastus externus is the incision of election.

There is one exception to this rule. A joint-body in the posterior part of the articulation cannot, owing to the narrow communication between the anterior and posterior portions of the capsule, commonly be removed by means of an anterior incision, unless one cuts the lateral ligaments. This is to be avoided. In such cases place your patient during the operative intervention in the ventral position and use a posterior incision. The length of the incision varies with the articulation and with the number and nature of the joint-body or bodies to be removed.

*Joint-lavage.*—Healthy joints do not call for irrigation. All irritation of joint-endothelium must be avoided. Irrigation of the peritoneal cavity as a prophylactic to peritonitis has been abandoned. Irrigation of the pleural cavity in the treatment of empyema is meddlesome; it has no curative value. Joint-irrigation is no preventive of arthritis. It waterlogs the tissues; it lowers tissue resistance; it serves no useful purpose; it delays recovery. We advise that it be not practised. We do not irrigate clean operative wounds. Why irrigate normal articulation?

*Joint-drainage.*—Joint-drainage frequently results in partial or complete ankylosis. A non-infected joint does not call for drainage.

*Immediate Closure of Articulation.*—Immediately after the removal of joint-mice, the joint-capsule and the divided overlying peri-articular tissues should be reunited. The synovial membrane and capsule are closed with catgut and the overlying tissues with removable non-absorbable suture material.

*Immobilization and Post-operative Treatment.*—In the cases under consideration, it is desirable, though not essential, that the operated joint be immobilized and that its articular surfaces be pulled apart. Secure fixation of the limb by the application of an anterior (rarely), posterior (generally) plaster of Paris splint moulded to the extremity. The splint should be of sufficient length to prevent all motion in the operated joint. To immobilize the knee-joint, the splint should extend from about two inches above the malleoli to within two inches of the perineum. By making extension on the distal segment of the limb one can secure the desirable separation of the articular surfaces. Thus the desired separation of the articular surfaces of the knee will be obtained by making moderate extension of the leg.

*Results.*—All the patients recovered from the operation.

In analyzing the results, we first note the important fact that there were no operative deaths. The case-reports show that in a large majority of cases non-operative treatment was tried and was found inappropriate, valueless, harmful. In the treatment of primary joint-bodies it is generally recognized that non-operative measures are not curative. They prolong the patient's local suffering and disability; they indirectly lead to, or aggravate existing articular changes. Repeated attacks of joint-locking usually lead to permanent hydrarthrosis. Chronic joint-effusions can disturb to an irremediable degree the anatomical integrity of joint-structures.

In the large majority of cases, over two hundred, joint-body removal was followed by restoration of anatomical and functional integrity, expressed as follows: Complete functional recovery, function of joint normal, perfect anatomical and functional recovery, full recovery, etc.

With such results before us no one should be allowed to go about suffering from loose or pedicled joint-bodies. Operation cures the condition and is followed in a large majority of cases, sooner or later, by complete anatomical and functional joint-integrity.

SUMMARY.

1. Primary joint-bodies occur in joints otherwise normal or presenting only such anatomical changes as are secondary to the presence of joint-body or bodies.

2. Primary joint-bodies occur in joints, the seat of congenital or acquired pathological states having no relation, either as cause or effect, to joint-mice.

3. Joint-bodies occur in the white and colored race, in both sexes and at all ages. They are met with maximal frequency in the male sex and during the third and fourth decades of life.

4. They are single or multiple, free or pedicled, and involve one or more similar or dissimilar joints. They may co-exist with extra-articular bodies and with various pathological peri- and extra-articular conditions.

5. Joint-bodies vary as to nature, shape, size, mobility, surface characteristics, and as to relation to articular bone ends and synovial membrane. All undergo, sooner or later, degenerative anatomical changes. All determine, sooner or later, degenerative anatomical changes in one or more or all the structures constituting the joint.

6. Joint-bodies, organized blood-clots excluded, are composed of one or more of the constituent tissues of the joint. In structure, they are of a fibrous, lipomatous, osseous, cartilaginous, osteo-cartilaginous or mixed nature. The joint-bodies reported were chips or fragments of bone, of cartilage, of bone and cartilage, masses of thickened indurated connective tissue, organized blood-clots, fibromata, lipomata, chondromata or osteomata.

7. Joint-bodies can co-exist with various articular lesions due to the same causative violence, or secondary either to joint-body irritation or to totally distinct and independent causes.

8. We know as to articulation involved that—

a. No diarthrodial joint is immune.

b. Excluding the joints of the upper extremity, the right and the left-sided joints are involved with about equal frequency.

c. The knee and the elbow are the most frequent seats of joint-bodies; joint-bodies in other articulations are clinical and pathological rarities.

d. Joint-bodies are found over five times as often in the knee as in all the other joints put together.

e. All bilateral cases reported in the literature are knee cases.

9. Violence is the first and foremost etiological factor. It may be direct (bumps, blows, falls, etc.), or indirect (torsions, efforts, sprains, strains, etc.), slight, moderate or severe, and cause, in addition to the joint-body, other articular and peri-articular injuries. In exceptional instances joint-bodies result from an inflammatory or a neoplastic process.

10. Cases of joint-bodies are not infrequently unrecognized, misdiagnosed, and, as a result, subjected to injudicious and, at times, actually injurious treatment.

11. Joint-body symptoms are referable to three factors:

a. *The Injury Causative of the Joint-body.*—The causative injury determines symptoms of acute articular and peri-articular joint inflammation, symptoms analogous to those occasioned by strains, sprains, contusions, fissures of cartilaginous articular surface and other joint traumatisms.

b. *The Joint-body Proper.*—Joint-bodies proper determine one, more or all of the following symptoms: Joint-pain and tenderness, joint-swelling, joint-crepitus, joint-effusion, joint-disability, joint-locking. These symptoms are merely suggestive of the condition which we are discussing. If the joint-body be palpated, the diagnosis is absolute. If the existence of the joint-body be demonstrated by the fluoroscope or skiagram, the diagnosis is manifest.

c. *The Secondary Joint-changes induced by the Presence of the Joint-body.*—The secondary articular changes induced by the presence of the joint-body cause symptoms varying from slight to complete joint-crippling. Repeated attacks of joint-locking and recurrent hydrarthrosis are responsible to a large degree for the deviations from the normal in contour, attitude and measurements of the affected articulation and also for the impairment of joint-function.

12. An attempt should always be made to diagnose not only the presence of joint-mice, but also their number, location, nature and other characteristics.

13. Roentgenography is an invaluable aid to diagnose the presence, number, location and many characteristics of joint-bodies. The X-ray examination should include an antero-posterior and a lateral view. Stereoscopic radiographs are to be preferred. Joint-bodies may exist though the X-ray plate be negative. If a joint-body from within be not the seat of calcific deposits or contain no osseous portion, it will cast no shadow upon the X-ray plate. X-ray findings have merely a confirmatory value.

14. The only relatively frequent condition which is difficult, at times, to differentiate from joint-bodies, is a partly or completely detached or ruptured semilunar cartilage. This condition also calls for an arthrotomy; therefore, the mistaking of a joint-body for a detached or ruptured semilunar cartilage, or vice versa, is not a significant diagnostic mistake.

15. Primary joint-bodies, irrespective of origin, location, nature, volume, number, mobility or surface characteristics invariably impair, sooner or later, the anatomical and functional integrity of the articulation which harbors them.

16. Joint-bodies should invariably be removed by an open operation, using that incision which gives best access to the joint-body or bodies and which inflicts the minimal amount of permanent injury upon the peri-articular structures. Chronic synovial effusions are dangerous to the integrity of the joint and justify the very slight risks which attend modern aseptic methods.

17. Important operative points are: (a) General anesthesia. (b) Use of an Esmarch constrictor which is removed immediately after ablating the joint-body and before suturing the capsular wound. (c) Incision parallel to the long axis of the limb. (d) Location and length of incision determined by the site, size and number of the joint-bodies. (e) Removal of joint-bodies by aid of instruments. Intra-articular manipulation should be reduced to a minimum. (f) No joint-irrigation. (g) No joint-drainage. (h) Separate suture of capsular and cutaneous wounds.

18. The removal of joint-bodies is an operation of great simplicity, having no morbidity and no mortality. If an arthrotomy be performed with aseptic precautions, the risks to the articulation, immediate or remote, are nil.

19. The removal of joint-bodies is followed by more or less complete anatomical and functional recovery. In cases of long standing, the return of complete functional integrity is not always immediate.

20. No one should be allowed to go about suffering from primary loose or pedicled joint-bodies. Operation cures the condition and in a large majority of cases leads to a complete return of anatomical and functional integrity.

